# A Chinese Herbal Decoction, Huoxue Qingyi Decoction, Promotes Rehabilitation of Patients with Severe Acute Pancreatitis: A Retrospective Study

**DOI:** 10.1155/2016/3456510

**Published:** 2016-03-24

**Authors:** Chao Hui Ji, Cheng Wu Tang, Wen Ming Feng, Ying Bao, Li Qin Yao

**Affiliations:** ^1^Department of Emergency Intensive Care Unit, First People's Hospital Affiliated to Huzhou University Medical College, Huzhou, Zhejiang 313000, China; ^2^Department of General Surgery, First People's Hospital Affiliated to Huzhou University Medical College, Huzhou, Zhejiang 313000, China

## Abstract

Severe acute pancreatitis (SAP) still remains an important surgical problem with high morbidity and mortality. The utilization of Traditional Chinese Medicine shows good prospects in therapy of SAP since it has advantages of more extensive pharmacological effects and fewer adverse effects. In this retrospective study, 38 patients received standardized treatment (control group) and 37 patients received Chinese herbal decoction, Huoxue Qingyi Decoction (HQD group), in addition to standard treatment for SAP. We found that the HQD group had a shorter hospital stay and lower initial expense than the control group (*P* < 0.05). The duration of hyperamylasemia and systemic inflammatory response syndrome (SIRS) were significantly shorter in HQD group (*P* < 0.05). The percentage of patients having any complication was much lower in HQD group than control group (27/38 versus 17/37, *P* < 0.05), especially pancreatic pseudocyst (10/38 versus 2/37, *P* < 0.05). No adverse effect induced by HQD was found. We concluded that the HQD was effective, safe, and economic for reduction of complication, for early recovery from systemic inflammation, and for promoting earlier rehabilitation from SAP.

## 1. Introduction

Severe acute pancreatitis (SAP) is a common cause of emergency hospital admission, with an increase in the incidence rate during the past 30 years. It can induce vascular leakage, shock, systemic inflammatory response syndrome, and even organ dysfunctions [1, 2]. Although many efforts have been contributed to the research in the disease, SAP is still dangerous and has many complications accompanied by a high mortality rate. Therefore, studies on improvement in therapy of SAP are urgently needed.

In contrast to western medicine, there is accumulating evidence suggestive of beneficial effects of plants used in Traditional Chinese Medicine (TCM) and compounds isolated from medicinal plants [3, 4]. The utilization of TCM shows good prospects in therapy of SAP since it has advantages of more extensive pharmacological effects and fewer adverse effects [5, 6]. The aim of the present retrospective study was to clarify the validity of the Chinese Herbal Decoction, Huoxue Qingyi Decoction (HQD), in patients with SAP.

## 2. Methodology

### 2.1. Patients

Between June 2010 and June 2014, 75 patients with a clinical diagnosis of SAP of whom 49 were men and 26 were women with a mean age of 45 years (range: 22 to 69 years) were admitted to the Department of Emergency Intensive Care Unit at First People's Hospital affiliated to Huzhou University Medical College (Zhejiang Province, China) within 3 days of disease onset. 38 patients received standardized medical therapy for SAP (control group) and 37 patients received additional HQD therapy (HQD group). Medical records were retrospectively reviewed.

The diagnostic and classification criteria for SAP were in accordance with the revised Atlanta criteria of 2013 [7], including two of the following features: (1) abdominal pain consistent with AP; (2) amylase activity at least three times greater than the upper limit of normal; and (3) characteristic findings of AP on contrast-enhanced computed tomography (CT) and, less commonly, on magnetic resonance imaging or transabdominal ultrasonography. Patients who had cardiac failure or pulmonary edema, were pregnant, underwent early surgical treatment or other TCM prior to hospital admission, or failed to give consent were excluded.

This study was conducted in accordance with the principles of the Declaration of Helsinki and “Good Clinical Practice” guidelines. Written informed consent was obtained from all patients. Approval of the study was obtained from the institutional review board.

### 2.2. Treatment

All of the patients received standardized medical therapy for SAP according to the United Kingdom, Chinese Medical Association, and International Association of Pancreatology guidelines [8–10]. In addition, patients in the HQD group received a 200 mL enema of HQD every 12 hours for 8 days and 50 mL of the same decoction either intragastrically or orally every 8 hours for 8 days.

The composition of HQD (shown in Table 1) consisted of* Salviae miltiorrhizae* 30 g,* Desmodium styracifolium* 30 g,* Rheum officinale* Baill 10 g, Natrii Sulfas 9 g,* Magnolia officinalis* 10 g, Radix Paeoniae Alba 10 g, Radix Bupleuri 10 g, Fructus Aurantii Immaturus 10 g, and* Scutellaria baicalensis* 10 g, which were purchased from Hangzhou Hu Qing Yu Tang Pharmaceutical Co., Ltd. (Hangzhou, China), whose products meet the commercial quality control according to the China Pharmacopoeia 2010. Herbal mixtures were made based on remedy menu by an experienced Chinese medicine practitioner.

### 2.3. Outcome Measurements

Clinical factors such as gender, age, body mass index (BMI), body weight, underlying comorbidity, etiological factor, severity of pancreatitis (Ranson's signs and Balthazar CT index), operability, hyperamylasemia duration, mechanical ventilation, mortality rate, hospital stay, initial hospitalization cost, and adverse effects were compared. All complications such as acute respiratory distress syndrome (ARDS), renal failure, hemorrhage, sepsis, pancreatic pseudocyst, and pancreatic abscess from admission to discharge from hospital were retrospectively analyzed. Respiratory failure was defined as the requirement for mechanical ventilation beyond 24 hours after surgery. ARDS and multiple organ failure were defined as per Bone et al. [11]. Sepsis required evidence of systemic inflammatory response syndrome (SIRS) with microbiological evidence of infection. SIRS was diagnosed by clinical manifestation of 2 or more of the following features: systolic blood pressure < 90 mmHg, tachycardia > 90/min, respiratory rate > 20/min or peripheral arterial CO_2_ tension (PaCO_2_) < 32 mmHg, temperature > 38.0°C or <36.0°C, leukocytosis > 12,000/*μ*L or leukopenia < 4000/*μ*L, or 10% immature (band) forms.

### 2.4. Statistical Analysis

All measurements were expressed as mean ± SD. The statistical analyses were performed using the two-sample *t*-test and adjusted Chi-square test for the two groups. The exact Chi-square test was also used if individual cell size was less than 5 counts. *P* value <0.05 was considered statistically significant.

## 3. Results

### 3.1. Patient Characteristics

There were no statistically significant differences between two groups in patient characteristics, in terms of gender, age, body mass index (BMI), severity of pancreatitis (Ranson's signs and Balthazar CT index), body weight, underlying comorbidity, and etiological factor (Table 2).

### 3.2. Clinical Outcomes

Clinical outcomes were shown in Table 3. The HQD group seemed to have had a shorter hospital stay and lower initial expense than the control group (*P* < 0.05). The duration of hyperamylasemia and SIRS was significantly shorter in HQD group (*P* < 0.05).

The percentage of patients having any complication was much higher in control group than HQD group (27/38 versus 17/37, *P* < 0.05) (Table 4), but there was no significant difference in in-hospital morbidity between two groups (Table 3). Further analysis about complications was performed and pancreatic pseudocyst was found significantly more frequent in control group compared with HQD group (*P* < 0.05) (Table 4). No adverse effect induced by HQD was found.

## 4. Discussion

Severe acute pancreatitis (SAP), a life-threatening condition characterized by edema, inflammation, hemorrhage, and necrosis of the pancreas, still remains an important surgical problem with high morbidity and mortality [12, 13]. Traditional Chinese Medicine active ingredients, such as emodin, magnolol, naringin, ginkgolide B, sanchinoside, taxol, resveratrol, rutoside, tetramethylpyrazine, and breviscapine, being used in treating SAP have advantages in not only acting on the pancreas, stomach, and intestines, but also having marked treatment effects on other visceral injuries arising from systemic inflammatory responses accompanying pancreatitis and blocking the disease progression [14].

Evidences in basic and clinical research suggest that it not only is an injury caused by the activated pancreatic enzymes but also involves pancreatic ischemia which leads to disturbance of pancreatic microcirculation that plays an important role in its pathophysiological processes [15]. The specific local microcirculatory changes cannot be prevented merely by adequate fluid therapy [16]. Salviae Miltiorrhizae, a traditional herbal medicine, has a low price and a wide range of clinical applications. It is proved to be effective in improvement of microcirculatory disturbances, elimination of oxygen free radicals, modulation of the metabolism of lipid inflammatory mediator, and blocking of calcium inflow and prevention of calcium overload [17]. Therefore, HQD in our study was a combination of Salviae Miltiorrhizae and Qingyi Decoction.

In this study, we found the patients of the HQD group showed a significantly shorter hospital stay, lower initial hospitalization cost, and shorter duration of SIRS and hyperamylasemia than control group. Though there was no significant difference in in-hospital morbidity between two groups, fewer complications especially pancreatic pseudocyst were found in HQD group than in control group. Notably, hemorrhage was not significantly alleviated in spite of antithrombotic properties of Salviae Miltiorrhizae.

Although Chinese herbal medicine is now widely used throughout the world, some herbal medicines have been associated with adverse effects and toxic effects [18]. Among the most serious adverse effects that can be caused by herbal medicine is drug induced liver injury (DILI). However, it was reported that many cases of DILI were induced by the self-purchase and administration of herb medicine, without a doctor's prescription [19]. Another study in Singapore found that 52.95% (9 of 17) of DILI cases were related to adulterated western medicine in herbal medicine [20]. Moreover, one study in China reported that only 24.2% of cases were related to herbal medicine and the other 75.8% to prescribed western medicine [21]. The current study showed that HQD did not induce any adverse effects including DILI.

In conclusion, HQD administrated per rectum and intragastrically or orally was well tolerable and did not increase the frequency of hemorrhage or adverse effects, at least in the short term. Therefore, application of HQD was effective, safe, and economic for reduction of complication, for early recovery from systemic inflammation, and for promoting earlier rehabilitation from SAP, though the precise mechanisms of therapeutic effects of HQD on patients required further exploration. Because of the retrospective nature and the small sample size, further prospective study with large sample size is needed to confirm the results of our study.

## Figures and Tables

**Table 1:** Components of Huoxue Qingyi Decoction with Latin and English names.

Name in Latin	Name in English	Dose
Salviae Miltiorrhizae	Danshen root	30 g
*Desmodium styracifolium*	Snowbell leaf tick clover herb	30 g
*Rheum officinale* Baill	*Rhubarb*	10 g
Natrii Sulfas	Glauber salt	9 g
*Magnolia officinalis*	Magnolia bark	10 g
Radix Paeoniae Alba	White peony root	10 g
Radix Bupleuri	Bupleurum root	10 g
Fructus Aurantii Immaturus	Immature bitter orange	10 g
*Scutellaria baicalensis*	Scutellaria root	10 g

**Table 2:** Patient characteristics.

	Control group (*n* = 38)	HQD group (*n* = 37)	*P* value
Gender			0.7438
Male	26	23	
Female	12	14	
Underlying comorbidity			
Hypertension	11	12	0.9388
Heart disease	3	2	0.9754
Diabetes mellitus	9	7	0.8245
Fatty liver	6	7	0.9578
Chronic obstructive pulmonary disease	3	4	0.9704
Etiological factor			0.8336
Biliary tract stone	17	20	
Hyperlipidemia	9	7	
Alcohol intake	10	9	
Other	2	1	
Age (year)	60.3 ± 7.1	61.8 ± 7.2	0.3667
Ranson's signs (score in 48 h)	4.9 ± 1.2	5.1 ± 1.1	0.4546
CT index (score in 48 h)	8.7 ± 2.1	7.9 ± 2.2	0.1115
BMI (kg/m^2^)	22.7 ± 3.3	23.2 ± 3.5	0.5263
Body weight (kg)	77.3 ± 9.4	76.2 ± 8.7	0.6008

**Table 3:** Clinical outcomes.

	Control group (*n* = 38)	HQD group (*n* = 37)	*P* value
Hyperamylasemia duration (day)	5.3 ± 2.1	3.7 ± 1.4	0.0001
Hospital stay (day)	16.1 ± 4.2	13.4 ± 5.7	0.0220
Mechanical ventilation	4	2	0.6953
Operability	2	1	0.9812
In-hospital mortality	1	0	0.9893
SIRS duration (day)	6.4 ± 2.2	4.5 ± 1.7	0.0004
Initial hospitalization cost (Chinese yuan)	34557.4 ± 1134.7	27446.7 ± 1375.6	0.0000

**Table 4:** Complications.

	Control group (*n* = 38)	HQD group (*n* = 37)	*P* value
Complication (%)	27 (71.1%)	17 (45.9%)	0.0485
Hemorrhage	3	1	0.6266
Pancreatic pseudocyst	10	2	0.0312
Pancreatic abscess	3	2	0.9514
Renal failure	7	4	0.5452
Heart failure	4	4	0.7382
Sepsis	12	8	0.9254
Acute respiratory distress syndrome	8	5	0.5773
